# Prevalence of Sexual Dysfunction in Patients With Chronic Prostatitis

**DOI:** 10.1155/ije/8530481

**Published:** 2025-10-29

**Authors:** Shahryar Zeighami, Sanaz Amiri, Leila Jamali, Alireza Shokrgozar, Javad Khalatbari, Iman Shamohammadi, Mehdi Nejat, Zahra Azadian, Fatemeh Azadian

**Affiliations:** ^1^Department of Urology, Shiraz University of Medical Sciences, Shiraz, Iran; ^2^Student Research Committee, Shiraz University of Medical Sciences, Shiraz, Iran; ^3^Shiraz University of Medical Sciences, Shiraz, Iran; ^4^Department of Clinical Health Psychology, Ka.C, Islamic Azad University, Karaj, Iran; ^5^Department of Psychological Capital, Department of Psychology, Tonekabon Islamic Azad University, Tonekabon, Iran; ^6^Fasa University of Medical Sciences, Fasa, Iran; ^7^Clinical Health Psychology, Kish International Branch, Islamic Azad University, Kish Island, Iran; ^8^Non-Communicable Diseases Research Center, Shiraz University of Medical Sciences, Shiraz, Iran

## Abstract

**Introduction:**

The prostatitis syndrome is one of the most common entities encountered in urologic practice. One of the most common problems that patients with chronic prostatitis typically face is sexual dysfunction. This study aimed to determine the prevalence of sexual dysfunction in patients with chronic prostatitis.

**Methods:**

The present study is a cross-sectional study on 400 patients aged 18 to 50 years who were treated for chronic prostatitis and for whom more than three months had passed since the onset of their prostatitis and treatment. The patients were included in the study through a census. The Changes in Sexual Functioning Questionnaire Short Form (CSFQ-14) was used to assess the sexual functioning status of the patients.

**Result:**

In this study, a total of 400 patients who had referred to the medical centers and hospitals affiliated with Shiraz University of Medical Sciences in 2024 due to chronic prostatitis were examined. The mean age of the patients was 40.83 ± 8.77. The mean sexual function score among patients with chronic prostatitis was 35.62 ± 8, with 89.2% of these individuals exhibiting signs of sexual dysfunction. Based on the results of this study, there was a significant relationship between sexual dysfunction and the type of relationship with the spouse (*p*=0.01).

**Conclusion:**

According to the findings of this study, sexual dysfunction is a common complication in men with chronic prostatitis. Most patients had a cold and weak relationship with their spouse.

## 1. Introduction

Prostatitis is a common urinary tract disease that many physicians find difficult to treat effectively. It is estimated that approximately half of all men will suffer from symptoms of prostatitis at some point in their lives. Prostatitis is the third most common urinary tract disease in men after benign prostatic hyperplasia (BPH) and prostate cancer [1].

Studies have shown that the prevalence of prostatitis is high, and it has a serious negative impact on patients' quality of life (QoL). Based on an epidemiological study of 12,743 participants, the prevalence of prostatitis-like symptoms in Chinese men is estimated to be approximately 8.4% [2]. Prostatitis accounts for approximately 25% of all office visits to urology clinics for genitourinary complaints worldwide. Unlike prostate cancer and BPH, which are predominantly diseases of older men, prostatitis affects men of all ages, with a particularly high incidence in the middle-aged age group. In Canada, approximately 9% of men experience symptoms of prostatitis during a year, of which 6% are problematic [3]. In more recent studies, the incidence of prostatitis has been shown to range from 3% to 16% in Europe, North America, and Asia, indicating that prostatitis is a significant global health problem [1].

Although prostatitis is not considered life-threatening, it presents a significant challenge for both physicians and patients [4]. Genitourinary and anorectal pain syndromes are relatively prevalent; however, effective treatments for this patient population remain elusive. Pain and functional impairment in these anatomical regions can be socially stigmatizing and may hinder patients' willingness to disclose their symptoms to healthcare providers [5]. Furthermore, most patients affected by prostatitis typically report a range of symptoms that extend beyond prostatitis itself, including lower urinary tract symptoms (LUTS) characterized by frequency, urgency, nocturia, dribbling, or a weak urinary stream; anorectal symptoms such as constipation, a sensation of foreign body presence in the rectum, and rectal pain during and after defecation; and external genital symptoms that may include genital pain or burning, premature ejaculation, spontaneous sexual arousal, or altered orgasm [6].

One of the most common problems that patients with chronic prostatitis typically face is sexual dysfunction. Sexual dysfunction is defined as a decrease or lack of sexual interest or desire, absence of sexual thoughts or fantasies, lack of responsive desire, and lack of motivation to achieve sexual arousal [7]. This definition is applicable to this sexual disorder in both men and women. Sexual activity can affect individuals' social health as well as their mental, emotional, and physical health. Sexual dysfunction can reduce an individual's QoL and self-esteem and harm their relationships with sexual partners [8]. The results of different studies have reported varying prevalence rates of sexual dysfunction in patients with prostatitis. A meta-analysis study showed that the prevalence of sexual dysfunction in patients with chronic prostatitis/chronic pelvic pain syndrome is 62% [9]. This figure has been reported to range from 33% to 92% in different studies [10, 11]. Therefore, it seems necessary to investigate the prevalence and treatment of sexual dysfunction in patients with prostatitis in addition to prostatitis treatment. The present study was conducted with the same aim, namely, to investigate the prevalence of sexual dysfunction in patients with chronic prostatitis in Fars Province (southern Iran).

## 2. Methods

The present study is a cross-sectional study conducted to investigate the prevalence of sexual dysfunction in patients with chronic prostatitis referred to the medical centers and hospitals affiliated with Shiraz University of Medical Sciences. In this study, 400 patients aged 18 to 50 years who were treated for chronic prostatitis and for whom more than three months had passed since the onset of their prostatitis and treatment were included in the study through a census. After obtaining informed consent from the patients to participate in the study, demographic information (age, occupation, level of education, smoking history, and drug use history) and sexual functioning status were obtained from the patients through an interview. The Changes in Sexual Functioning Questionnaire Short Form (CSFQ-14) was used to assess the sexual functioning status of the patients. The Sexual Dysfunction Questionnaire was designed by Clare M.C. Carvey and Clayton in 2006 and has 14 questions that measure four subscales of desire, arousal, orgasm, and pleasure. The scoring of this test is based on a 5-point Likert scale (1 = *never* and 5 = *always*). The sum of all questions indicates the score of this test, which ranges from 14 to 70. High scores indicate better sexual performance, and low scores indicate sexual dysfunction [1]. The validity and reliability of this questionnaire have been confirmed in Iranian samples, and in the study by Mohammad Ali et al. (2020), the face and content validity of this scale were confirmed by university professors. Cronbach's alpha method was used to examine the validity of the scale, and the value of this coefficient was 0.88 [12]. In this study, the cutoff points for the total score of the CSFQ-14, which indicates sexual dysfunction, was considered to be 47.0 or less. Also, the total CSFQ-14 scores are grouped into three categories based on severity: “*mild*” (42–47), “*moderate*” (35–41), and “*severe*” (< 35) [13]. At the conclusion of the study, the data were entered into SPSS Version 16 for analysis, utilizing chi-square tests and independent *t*-tests for statistical evaluation.

The aforementioned study design received approval from the Ethics Committee of Shiraz University of Medical Sciences, under code IR.SUMS.MED.REC.1402.444.

## 3. Results

The present study is a cross-sectional study conducted to investigate the prevalence of sexual dysfunction in patients with chronic prostatitis. In this study, a total of 400 patients who had referred to the medical centers and hospitals affiliated with Shiraz University of Medical Sciences in 2024 due to chronic prostatitis were examined. The mean age of the patients was 40.83 ± 8.77. Other demographic variables of the patients are listed in Table 1.

The results of Table 1 show that almost all 8.99% of the patients were married, most of them (78%) had a family relationship with their spouse, and their education and that of their spouse was high school (37% and 32%). In terms of employment status, most of them were employees or workers (63%), and their relationship with their spouse was usually cold (32%), but most of them (88%) had not thought about divorce so far, and most of them (69%) had 2 or fewer children.

The results of Table 2 show that most patients with chronic prostatitis did not have concomitant neurological diseases (81%) and did not have other diseases such as blood pressure, diabetes, or thyroid. Most of them also did not use drugs, cigarettes, or hookah (80%).

The results of Table 3 show that among patients with chronic prostatitis, only 10.8% have normal sexual function. This means that a small proportion of patients are without sexual dysfunction. In contrast, 15.3% have mild dysfunction, 27.5% have moderate dysfunction, and 46.5% have severe dysfunction. Overall, more than three-quarters of patients experience some degree of sexual dysfunction, and almost half of them have severe dysfunction.

The data presented in Table 4 indicate that the mean sexual function score among patients with chronic prostatitis was 35.62 ± 8, with 89.2% of these individuals exhibiting signs of sexual dysfunction.

Based on the results of Table 5, there was no relationship between sexual dysfunction and marital status, family relationship with spouse, individual education, spouse's education, employment status, and number of children (*p* > 0.05). However, there was a significant relationship between sexual dysfunction and the type of relationship with the spouse (*p*=0.01). The greatest difference was in the intimate, respectful, and indifferent relationship with the spouse. Thus, patients who did not have sexual dysfunction had an intimate relationship with their spouse in 18.2% of cases. While in patients who had sexual dysfunction, they had an intimate relationship with their spouse in only 5.3% of cases. Also, patients with sexual dysfunction had respectful behavior in 26.1% and indifferent behavior in 13.7% of cases toward their spouse, while these percentages were 16.3% and 7% in patients who did not have sexual dysfunction.

Most patients had a cold and weak relationship with their spouse. Also, the average age of patients with chronic prostatitis who had sexual dysfunction was 41.17 ± 8.71, and in those who had normal sexual function, it was 39.14 ± 8.49. The average age of patients who had sexual dysfunction was significantly higher than that of patients who had normal sexual function (*p*=0.054).

The results of Table 6 show that there is no relationship between sexual dysfunction and neurological diseases, diabetes, thyroid, drug use, smoking, and hookah. However, sexual dysfunction was associated with high blood pressure (*p*=0.02).

## 4. Discussion and Conclusion

This study aimed to investigate the prevalence of sexual dysfunction in patients with chronic prostatitis. The findings indicate that 89.2% of patients suffering from chronic prostatitis also experienced sexual dysfunction. These results align with those of previous studies. A meta-analysis revealed that the prevalence of sexual dysfunction in patients with chronic prostatitis/chronic pelvic pain syndrome was 62% [9]. Additionally, the research by Vittorio Magri et al. found that among 614 patients with chronic bacterial prostatitis, erectile dysfunction was present in 49.8%, while premature ejaculation was observed in 40.7%. When considering other disorders related to ejaculation, orgasm, and libido, at least one form of sexual dysfunction was identified in 86.3% of the cohort [14]. Kumsar et al. reported that in a study involving 138 patients with prostatitis, the mean score on the International Index of Erectile Function-5 was significantly lower in the prostatitis group compared to the nonprostatitis group [15]. Furthermore, Liang et al. assessed 2000 patients with chronic prostatitis and found that 49% exhibited comorbid sexual dysfunction, with 26.4% experiencing premature ejaculation, 14.9% having erectile dysfunction, and 7.7% reporting both conditions [16]. Some researchers have highlighted the influence of chronic pain, stress, and depression associated with chronic prostatitis/chronic pelvic pain syndrome on the development of sexual dysfunction in these patients. However, data from other studies suggest that sexual dysfunction may occur independently of psychological distress [17]. Another contributing factor could be the catastrophizing of pain. Research indicates that when individuals experience pain primarily in the pelvic region, they may exaggerate the severity of their pain and ruminate on its impact on their daily lives and sexual functioning. This preoccupation with the negative repercussions of impaired functioning often leads to adverse emotions and further performance decline [18]. Given the high prevalence of sexual dysfunction among patients with prostatitis, it is essential to consider specific and targeted treatments for sexual dysfunction—such as physical therapy, psychological therapy, and lifestyle modifications—concurrently with prostatitis treatment.

The present study's results indicate that patients with sexual dysfunction experience a weaker relationship with their spouse compared to those without sexual dysfunction. This finding is consistent with various studies. For example, the research conducted in Brazil involving 100 heterosexual couples demonstrated a strong association between sexual dysfunction and relationship dissatisfaction, as well as a positive correlation between depressive symptoms and marital dissatisfaction [19]. Additionally, a study by Manjula et al. highlighted the close relationship between sexual dysfunction and marital distress, emphasizing the significant role of sexual interaction and communication in marital quality and intimacy [20]. Sexual dysfunction markedly impacts interpersonal functioning and overall QoL for both men and women, with marital quality being largely dependent on couples' satisfaction with their sexual relationships. Furthermore, sexual function is recognized as a crucial element of intimacy. Ferreira et al. found that intimate emotional connection and self-integration are vital for fostering sexual desire within marriage or long-term relationships [21]. Individuals with hypoactive sexual desire disorder (HSDD) exhibit lower levels of intimacy, satisfaction, and quality of intimacy compared to controls. It has been reported that women often engage in sexual relationships due to the emotional intimacy and enhanced well-being they provide, which they perceive as rewarding [22]. Moreover, the quality of communication between partners has been identified as a critical factor influencing relationship satisfaction.

## 5. Conclusion

According to the findings of this study, sexual dysfunction is a common complication in men with chronic prostatitis. Most patients with chronic prostatitis had a cold and weak relationship with their spouse which can be caused by sexual dysfunction in them.

### 5.1. Strengths

This study had a suitable sample size and, by focusing on a specific population (prostatitis patients), can better examine the relationship between prostatitis and sexual dysfunction. The findings of this study can also help physicians better manage patients with prostatitis and related sexual problems.

### 5.2. Limitations

The limitations of this study include the lack of a control group (e.g., healthy men or patients with other conditions). Also, because the study was conducted as a cross-sectional study, a causal relationship between prostatitis and sexual dysfunction cannot be proven. It is recommended that future studies use the NIH-CPSI or a validated tool to classify the severity of prostatitis.

## Figures and Tables

**Table 1:** Demographic characteristics of patients with chronic prostatitis in the present study.

Variable		Number	Percent
Marital status	Married	399	99/8
	Single	1	0.02

Family relationship with spouse	Yes	88	22
	No	312	78

Education	Illiteracy	38	9.5
	Elementary	98	24.5
	High school	148	37
	University	116	29

Spouse's education	Illiteracy	62	15.5
	Elementary	90	22.5
	High school	129	32.3
	University	119	29.8

Employment status	Government job	130	32.5
	Unemployed	45	11.3
	Worker	133	33.3
	Driver	61	15.3
	Farmer	31	7.8

Relationship type	Close	27	6.8
	Friendly	92	23
	Respectful	100	25
	Indifferent	52	13
	Cold	129	32.3

Have you ever contemplated pursuing a divorce?	Yes	47	11.8
	No	353	88.3

Number of children	0–2	276	69.2
	3–5	114	28.5
	More than 5	10	2.3

**Table 2:** Characteristics of behavioral encounters and comorbidities of patients with chronic prostatitis in the present study.

Type of disease/exposure		Number	Percent
Neurological diseases	Yes	76	19
	No	324	81

High blood pressure	Yes	79	19.8
	No	321	80.3

Diabetes	Yes	39	9.8
	No	361	90.2

Thyroid disorder	Yes	16	4
	No	384	96

Drug use	Yes	65	16.3
	No	355	83.8

Cigarettes	Yes	82	20.5
	No	318	79.5

Hookah	Yes	78	19.5
	No	322	80.5

**Table 3:** Distribution of patients with chronic prostatitis based on the severity of sexual dysfunction.

Variable	Percent	Number
Normal sexual function	43	10.8
Mild sexual dysfunction	61	15.3
Moderate sexual dysfunction	110	27.5
Severe sexual dysfunction	186	46.5

**Table 4:** Mean and standard deviation of sexual function scores in patients with chronic prostatitis and their dimensions.

Variable	Mean	SD	Maximum	Minimum
Sexual function	35.62	8	59	14
Sexual function (desire dimension)	10.98	2.79	20	5
Sexual function (arousal dimension)	12.68	3.54	23	4
Sexual function (pleasure dimension)	7.40	2.44	14	3
Sexual function (orgasm dimension)	4.55	1.72	9	2

**Table 5:** Association between demographic variables and sexual dysfunction in patients with chronic prostatitis.

Variable		Normal sexual function	Sexual dysfunction	*p* value^∗^
		*N* (%)	*N* (%)	
Marital status	Married	43 (100)	356 (99.7)	0.78
	Single	0 (0)	1 (0.3)	

Family relationship to spouse	Yes	8 (18.6)	80 (22.4)	0.97
	No	35 (81.4)	277 (77.6)	

Education	Illiterate	5 (11.6)	33 (9.2)	0.74
	Elementary	12 (27.9)	96 (24.1)	
	High school	14 (32.6)	134 (37.5)	
	University	12 (27.9)	104 (29.1)	

Spouse's education	Illiterate	8 (18.6)	54 (15.1)	0.45
	Elementary	6 (14)	84 (23.5)	
	High school	16 (37.2)	113 (31.7)	
	University	13 (30.2)	106 (29.7)	

Job type	Government job	15 (34.9)	115 (32.2)	0.59
	Unemployed	4 (9.3)	41 (11.5)	
	Worker	15 (34.9)	118 (33.1)	
	Driver	6 (14)	55 (15.4)	
	Farmer	3 (7)	28 (7.8)	

Relationship type	Sincerely	8 (18.2)	19 (5.3)	0.01
	Friendly	11 (25.6)	81 (22.7)	
	Respectful	7 (16.3)	93 (26.1)	
	Indifferent	3 (7)	49 (13.7)	
	Cold	14 (32.6)	115 (32.2)	

Decision to divorce	Yes	4 (9.3)	43 (12)	0.44
	No	39 (90.7)	314 (88)	

Number of children	0–2	33 (76.7)	254 (71.1)	0.31
	3–5	10 (23.2)	76 (21.2)	
	More than 5	0	27 (7.5)	

^∗^ *p* for chi-square test.

**Table 6:** Relationship between behavioral exposure variables and diseases associated with sexual dysfunction in patients with chronic prostatitis.

Type of disease/exposure		Normal sexual function	Sexual dysfunction	*p* value
		*N* (%)	*N* (%)	
Neurological diseases	Yes	4 (9.3)	72 (20.2)	0.25
	No	39 (90.7)	285 (79.8)	

High blood pressure	Yes	3 (7)	76 (21.3)	0.02
	No	40 (93)	281 (78.7)	

Diabetes	Yes	4 (9.3)	35 (9.8)	0.20
	No	39 (88.7)	322 (90.2)	

Thyroid disorders	Yes	1 (2.3)	15 (4.2)	0.27
	No	42 (97.7)	342 (98.7)	

Drug use	Yes	7 (16.3)	58 (16.2)	0.83
	No	36 (83.7)	298 (83.8)	

Cigarettes	Yes	8 (18.6)	73 (20.4)	0.79
	No	35 (81.4)	284 (79.6)	

Hookah	Yes	8 (18.6)	70 (19.6)	0.71
	No	35 (81.4)	287 (80.4)	

## Data Availability

Data supporting the findings of this study are available from the corresponding author upon reasonable and justified request.
